# Mice Mutated in the Third Fibronectin Domain of L1 Show Enhanced Hippocampal Neuronal Cell Death, Astrogliosis and Alterations in Behavior

**DOI:** 10.3390/biom13050776

**Published:** 2023-04-29

**Authors:** Ludovica Congiu, Viviana Granato, Igor Jakovcevski, Ralf Kleene, Luciana Fernandes, Sandra Freitag, Matthias Kneussel, Melitta Schachner, Gabriele Loers

**Affiliations:** 1 Zentrum für Molekulare Neurobiologie, Universitätsklinikum Hamburg-Eppendorf, Falkenried 94, 20251 Hamburg, Germanyralf.kleene@zmnh.uni-hamburg.de (R.K.); sandra.freitag@zmnh.uni-hamburg.de (S.F.); matthias.kneussel@zmnh.uni-hamburg.de (M.K.); 2 Institut für Anatomie und Klinische Morphologie, Universität Witten/Herdecke, 58455 Witten, Germany; igor.jakovcevski@uni-wh.de; 3 Keck Center for Collaborative Neuroscience, Department of Cell Biology and Neuroscience, Rutgers University, Piscataway, NJ 08554, USA

**Keywords:** L1CAM, hippocampus, neuronal cell death, astrogliosis, behavior

## Abstract

Adhesion molecules play major roles in cell proliferation, migration, survival, neurite outgrowth and synapse formation during nervous system development and in adulthood. The neural cell adhesion molecule L1 contributes to these functions during development and in synapse formation and synaptic plasticity after trauma in adulthood. Mutations of L1 in humans result in L1 syndrome, which is associated with mild-to-severe brain malformations and mental disabilities. Furthermore, mutations in the extracellular domain were shown to cause a severe phenotype more often than mutations in the intracellular domain. To explore the outcome of a mutation in the extracellular domain, we generated mice with disruption of the dibasic sequences RK and KR that localize to position _858_RKHSKR_863_ in the third fibronectin type III domain of murine L1. These mice exhibit alterations in exploratory behavior and enhanced marble burying activity. Mutant mice display higher numbers of caspase 3-positive neurons, a reduced number of principle neurons in the hippocampus, and an enhanced number of glial cells. Experiments suggest that disruption of the dibasic sequence in L1 results in subtle impairments in brain structure and functions leading to obsessive-like behavior in males and reduced anxiety in females.

## 1. Introduction

The cell adhesion molecule L1, a member of the immunoglobulin superfamily, is essential for nervous system development, synaptic functions and regeneration after injury [1,2]. In mammals, L1 is expressed in the central nervous system on subsets of developing and differentiated neurons, e.g., on cerebellar, cortical and hippocampal neurons and on immature oligodendrocytes. On differentiated neurons, L1 is mainly found at contact sites between axons and on growth cones [3]. L1 is involved in axon guidance and fasciculation [4,5], neurite outgrowth [6], neuronal cell migration and differentiation [7,8,9] as well as synapse formation [10] and synaptic plasticity [11,12,13]. These functions not only depend on L1′s interactions via its extracellular or intracellular domain with various binding partners and activation of signaling cascades, but also on proteolytic cleavage of L1 by plasmin [14], PC5A [15], cathepsin E [16], myelin basic protein [17] and metalloproteinases [18,19]. The resulting intracellular and extracellular L1 fragments with or without the transmembrane domain were shown to be essential for several L1 functions, such as neurite outgrowth and neuronal survival [16,17], myelination [18], synaptic plasticity [12] and regeneration after injury [20]. Furthermore, cleavage of L1 is also important for tumor cell invasion and motility [21,22,23]. L1 fragments were found to be shed into the extracellular space and transported into intracellular compartments as mitochondria and the nucleus [12,24,25]. The importance of L1 and its fragments in nervous system development is underscored by the finding that mutations of L1 in humans result in L1 syndrome, which is associated with mild-to-severe brain malformations, mental disabilities and spasticity [26,27]. Mice deficient in L1 or carrying mutations found in L1 syndrome patients are impaired in motor coordination [11,26] and also exhibit brain malformations, which are features of the X-chromosome-linked L1 syndrome [28,29,30,31]. Spatial learning abilities and L1 levels are reduced in rats after X-irradiation at an embryonic stage [32]. L1 heterozygous female mice exhibit reduced social behaviors and excessive self-grooming [33]. In contrast, mice ectopically expressing L1 by astrocytes showed increased behavioral flexibility and selectivity during learning and relearning [11]. These studies confirm that L1 expression and signaling are important for normal brain development and brain functions, but do not shed light on the functions of L1 fragments and the importance of L1 proteolysis for brain development and nervous system functions in vivo. To evaluate these functions, we have generated gene-edited mice expressing L1 with mutated dibasic sequences in the third fibronectin type III homologous (FNIII) domain at position 858–863 (exchanging wild-type RKHSKR for mutant SKHSSS, hereafter termed L1/858–863 or mutant mice), thereby disrupting the plasmin, PC5a and trypsin cleavage site (Supplementary Figure S1). Here, we found that L1/858–863 mice exhibit enhanced numbers of caspase-3 positive cells and astrogliosis in the hippocampus, with lower numbers of pyramidal cells in the CA3 subfield. L1/858–863 mice display changes in behavior such as reduced anxiety and enhanced burying behavior suggesting that the third FNIII domain of L1 is important for L1′s functions.

## 2. Materials and Methods

### 2.1. Mice

Gene-edited mice expressing murine L1 with a mutation of the dibasic sequences within RKHSKR to SKHSSS at position 858–863 in the third FNIII domain (L1/858–863 mutant mice) have been described [34]. Mice were bred and maintained at the Universitätsklinikum Hamburg-Eppendorf on a 12 h light/12 h dark cycle and maintained under standard housing conditions (21 ± 1 °C, 40–50% humidity, food and water ad libitum). Two-to-five-month-old transgenic L1/858–863 and WT males or females were used for all experiments. Experiments were approved by the Behörde für Justiz und Verbraucherschutz of the State of Hamburg (animal permit number N 073/2020 (approval date 16 September 2020)) and experiments were designed, and the manuscript was prepared according the ARRIVE guidelines [35].

### 2.2. Reagents

Reagents were from Sigma-Adrich (Taufkirchen, Germany) or Carl Roth (Karlsruhe, Germany) if not indicated otherwise.

### 2.3. Histology

One day after completion of the behavioral studies, animals were deeply anesthetized by intraperitoneal injections of ketamine and xylazine (80 mg Ketanest^®^ (Pfizer Pharma PFE GmbH, Berlin, Germany) and 10 mg Xylazine^®^ (WDT, Garbsen, Germany), per kg body weight) and then transcardially perfused with fixative (4% *w*/*v* paraformaldehyde and 0.1% *w*/*v* CaCl_2_ in phosphate buffered saline (PBS), pH 7.4). Brains were extracted and postfixed for at least 24 h at 4 °C in the same fixative. Tissue was then immersed in a 15% *w*/*v* sucrose solution in PBS, pH 7.4, for 2 days at 4 °C and then in 30% *w*/*v* sucrose solution in PBS, pH 7.4, for 2 days at 4 °C. Then, brains were embedded in Tissue Tek (Sakura Finetek, Umkirch, Germany), frozen by a 2-min immersion into 2-methylbutane precooled to −80 °C and stored at −80 °C until sectioned. Brains were cut into 40 µm-thick coronal sections in a cryostat (Cryostar NX70; ThermoFisher Scientific, Waltham, MA, USA) and collected in section storage buffer (0.02% *w*/*v* sodium azide in PBS) and stored at 4 °C.

Free-floating cryosections from the brains of three or four male mice per genotype were mounted on glass slides (Superfrost Plus, ThermoFisher Scientific), air dried and stained with cresyl violet and Luxol Fast Blue to visualize neurons and myelin [29] or used for immunohistochemistry. Staining was performed with Luxol Fast Blue solution (0.1% *w*/*v* Luxol Fast Blue, 95% ethanol and 0.5% glacial acetic acid in distilled water) at 57 °C overnight. Sections were then washed in 95% ethanol and distilled water before being stained in a 0.05% *w*/*v* lithium carbonate solution for 3 min. The differentiation was then continued with 70% ethanol and distilled water. Afterwards, sections were stained in a cresyl violet solution (0.1% *w*/*v* cresyl violet acetate in water) at 57 °C for 5–10 min. Finally, sections were differentiated with several changes of 95% ethanol, dehydrated in absolute ethanol, cleared in xylene and mounted with Eukitt quick-hardening mounting medium (Fluka, Buchs, Switzerland). Image acquisition was performed using a Zeiss AxioObserver or Apotome 3 microscope (Carl Zeiss) with a 10× objective (aperture 0.3), and thickness and areas were counted using ImageJ software (version 1.53; https://imagej.nih.gov/ij/index.html; RRID:SCR_003070; accessed date 9 February 2023; [36]). For analysis of corpus callosum thickness, hippocampal area and ventricle size sections from four mice per genotype were used. Corpus callosum thickness (vertical dimension) was determined at Bregma levels −2.18, −0.46, −0.26 and 0.74. Ventricle size was measured at two different Bregma levels (0.74, −0.34) and calculated relative to the entire area of the section.

### 2.4. Immunohistochemistry

Cryosections from three mice per genotype and sex were left at the room temperature (RT) to dry for 2 h. After a brief wash in PBS, the sections were incubated in a blocking solution containing 5% normal goat serum and 0.2% Triton X-100 dissolved in PBS for 1 h at RT to block non-specific binding. Afterwards, the sections were incubated overnight with the primary antibody, diluted in blocking solution, at 4 °C. The following primary antibodies were used: anti-neuron specific nuclear antigen (NeuN, mouse monoclonal, clone A60, 1:1000; Sigma-Aldrich), anti-glial fibrillary acidic protein (GFAP, rabbit polyclonal, 1:500; DakoCytomation, Hamburg, Germany), anti-Iba1 (rabbit polyclonal, 1:1500; Wako Chemicals, Neuss, Germany), and anti-caspase-3 active (rabbit polyclonal, 1:2000; R&D Systems, Wiesbaden, Germany). After washing in PBS (3 × 15 min at RT), the appropriate Cy2- or Cy3-conjugated secondary antibody (Jackson ImmunoResearch, Cambridgeshire, UK) diluted 1:200 in PBS was applied for 2 h at RT. After subsequent washes in PBS (3 × 15 min at RT), the sections were mounted in anti-quenching medium (RotiMount with DAPI; Carl Roth) and stored in the dark at 4 °C.

### 2.5. Stereological Analyses

To estimate cell densities (number of cells per volume) optical dissector method was used, as described [37]. The counts were performed directly under an Axioskop microscope (Zeiss, Oberkochen, Germany) equipped with a motorized stage and Neurolucida software-controlled computer system (MicroBrightField Europe, Magdeburg, Germany). Cell densities were estimated in every 10th spaced serial section (250 μm apart) in which the areas of interest were seen. Counting was based on the identification of the position of the cell nuclei of immunostained cells within the dissector using a 40× objective. The parameters for the stereological analysis were as follows: guard space depth 2 μm, base and height of the dissector were 3600 μm^2^ and 10 μm, respectively, distance between the optical dissectors 60 μm and the objective was a Plan-Neofluar^®^ 40×/0.75 (Zeiss, Oberkochen, Germany). The same parameters were used for counting the nuclei in the pyramidal layer except for the base of the dissector and in the space between dissectors, which were 625 μm^2^ and 25 μm, respectively. Left and right cortical and hippocampal areas were evaluated. All results shown are averaged bilateral values. As the activated caspase 3 positive cells were very rare, they were counted per whole hippocampus section.

### 2.6. Behavior

For behavioral studies, mice housed in groups of three to six mice were accustomed to an inverted day–night cycle (light off at 7:00 a.m.) for one week and then to the experimenter by handling for one hour per day for one week. Before the experiments mice were transported to the experimental room next to the vivarium which was illuminated by dim red light and left for 5–10 min for habituation. Tests started and ended at least 2 h after light offset and 3 h before light onset, respectively. All the mazes used for behavioral analyses were cleaned between mice with soap and water and then with 30% ethanol. Care was taken to minimize pain or discomfort for the animals. Tracks representing the position of the mice were created and analyzed with EthoVision (Noldus, Wageningen, The Netherlands; https://www.noldus.com/ethovision; RRID:SCR_000441; access date 7 March 2023) [38]. Manual scoring of behavior was performed by a trained experimenter blinded to the genotype of the mice using The Observer software (Noldus). Numbers of mice used per group are indicated in the figure legends. The timeline of the experiments is presented in Supplementary Figure S7.

#### 2.6.1. Open Field and Elevated plus Maze

To evaluate activity levels as well as exploratory and anxiety-like behavior, mice were evaluated in the open filed and elevated plus maze [39]. A multiple unit open field (OF) consisting of four activity chambers was used. Each OF chamber measured 50 cm (length) × 50 cm (width) × 50 cm (height), was made from white high density and non-porous plastic and was illuminated with 50 lux. A mouse was placed in an acrylic glass cylinder located in one corner of each activity chamber. When the cylinder was lifted, the mice could freely move in the arena for 20 or 30 min and the distance moved and behavior in the arena was recorded. In addition to the time spent in the center of the arena, the total distance moved, the average distance from the wall and the frequency to enter the center zone, which were analyzed for 20–30 min, the following specific behavior parameters were analyzed only for the first 5 min of the test: rearing on the wall (vertical exploration by standing on the back paws with one or two forepaws touching the wall), rearing the off wall and self-grooming. 

The elevated plus maze consisted of a plus shaped arena with four arms, 30 cm long and 5 cm wide, connected with a center zone of 5 × 5 cm. Two opposing arms were bordered with a 2 mm rim (open arm), whereas the other two had a 15 cm high wall (closed arm). The maze was elevated 75 cm from the floor and illuminated with 10 lux. Mice were placed in the center of the maze facing the open arm and observed for 5 min. The following parameters were analyzed: open and closed arm entries (calculated when all the four paws were inside the arm), stretch-attend posture, calculated when the mouse stretched forward and retracted to the original position without forward locomotion, self-grooming and head dips.

#### 2.6.2. Social Interaction

Motivation to investigate a social stimulus was tested by giving the experimental mouse the choice to investigate an unfamiliar mouse or a familiar sex-matched mouse [38]. The arena used for the open field test (50 × 50 cm) was divided into two identical compartments by a 40 cm high wall with an opening in the middle allowing the mouse access to both the compartments. A cylinder with a metal grid mesh allowing olfactory exploration between mice was located in one corner of each compartment containing either a familiar or an unfamiliar mouse. Familiar mice were recruited from heterozygous siblings living in the same cage as the experimental mice. Unfamiliar mice were heterozygous or WT mice from different cages that were not used as subjects in the behavior tests. First the familiar and unfamiliar mice were placed under the cylinders and then the subject mouse was placed in the arena and left free to move between compartments for 20 min. The room was illuminated with 5 lux during recording of the behavior and distance moved, time spent in each compartment and time spent in proximity of the two cylinders were determined. The times spent in the proximity of the familiar and unfamiliar mouse were used to calculate the preference index. The time spent exploring the unfamiliar mouse was divided by the time exploring the familiar and unfamiliar mouse. Values higher than 0.5 indicate a preference to explore the unfamiliar mouse.

#### 2.6.3. Circadian Activity

Mice were housed singly in a cage with a size of 23 × 20 × 15 cm for 3 days and then the circadian activity during a 24 h day–night cycle was recorded with a motion detector (Mouse-E-Motion; Infa-E-Motion, Hamburg, Germany) that was placed on top of the cage. The activity of the mice was recorded in 4 min time bins and E-motion software was used to analyze the activity of the mice [38].

#### 2.6.4. Rotarod Test

To evaluate motor coordination and motor learning of mice, the rotarod and pole test were used. For the rotarod test [40,41], mice were trained on a rotating rod with 3 cm diameter (Rota-rod for mice, UGO BASILE S.R.L., Germany) for two days with two trials with constant speed (4 rpm) for 2 min and three trials of training with acceleration (4–40 rpm) for 4 min. After every training trial, the training was stopped for an interval of 10 min before continuing with the next training phase. For the next three to four consecutive days mice were subjected to the test: mice were located on the 3 cm diameter rod, with acceleration from 4 to 40 rpm within 300 s (5 min). The tests were performed with red light and the latency to fall from the rod was recorded.

#### 2.6.5. Beam Walking

Motor coordination and balance were evaluated also by the beam walking test. Mice were trained to cross a wooden beam (90 cm long and 5 cm wide, positioned 50 cm from the ground) to reach their home cage at the end of the beam. To further motivate the mice, food was placed at the end of the beam. During the trial, mice were recorded from behind while walking on the beam, from the experimenter to the home cage, and the time to cross the bream, the heal-tail angle and the foot-base angle were determined [42]. Experiments were performed under 50 lux light.

#### 2.6.6. Grip Strength

Strength of the forelimbs, as measure for neuromuscular function, was measured using the GripStrengthMeter system (TSE Systems, Berlin, Germany; version 303500-M+R/SW). Mice were suspended by the tail and allowed to grasp with the front paws a stainless-steel grip attached to a dynamometer. The maximal force applied while pulling the mice until they released the grip was recorded. Mice were tested in three sessions of three trials. The mean of the maximal value among three trials was used for analysis [43].

#### 2.6.7. Pole Test

In the pole test, motor coordination was monitored while mice were climbing down a pole. The test was performed as described [38]. Mice were placed head upward at the top end of a rough-surfaced vertical wooden rod (48.5 cm long, 3 mm diameter). The time needed by the mice to reach the floor with all four paws and the manner by which mice descended from the top of the pole were analyzed.

#### 2.6.8. Marble Burying

To investigate if L1/858–863 mice show repetitive, compulsive-like behaviors, the marble burying test was used [44,45]. The test was performed under red light and in a 42 × 24 × 12 cm cage. A 5 cm layer of fresh bedding material covered the floor of the cage, and 20 black marbles (diameter 1.5 cm) were placed on top of the bedding. The mice were placed in one corner of the cage and left for 30 min to explore and move before being returned to the home-cage. Numbers of buried marbles were counted and scored. The criterion to count marbles as buried was that they were covered at least 50% by bedding.

### 2.7. Statistics

All numerical data are presented as single values and group mean values with standard error of the mean (SEM) or as group mean values with standard deviation (SD). Normal distribution of the data was determined using Shapiro–Wilk test and Levene’s test before choosing the appropriate statistical test. Statistical tests used for comparisons are indicated in the figure legends. Analyses were performed using the SPSS or GraphPad software.

## 3. Results

### 3.1. Behavioral Analysis

#### 3.1.1. Body Weight and Grip Strength

We first determined the body weight and grip strength of mice to rule out that differences in behavior observed are due to altered body weight or strength of mice. No difference in the appearance of L1/858–863 (mutant) mice in comparison to the wildtype (WT) mice was observed when mice were examined starting from postnatal day 21 till 5 months of age. Regarding the weight, we observed two-month-old WT males 27.17 ± 0.74 g, mutant males 27.20 ± 2.18 g, WT females 25.83 ± 1.80 g and mutant females 22.70 ± 1.08 g and five-month-old WT males 31.47 ± 2.62 g, mutant males 32.18 ± 3.99 g, WT females 23.38 ± 1.43 g and mutant females 23.47 ± 2.17 g (average weight ± SD). Grip strength of WT and mutant mice was similar (grip strength in g-force for WT males 25.2 ± 1.73 g, for mutant males 25.42 ± 1.50 g, for WT females 23.89 ± 1.41 g, and for mutant females 23.76 ± 1.32 g). When evaluating quadriceps muscle function in the beam walking test, mutant and WT mice needed a similar time to cross the beam and plantar stepping abilities of mutant mice were not different compared to the WT mice (Supplementary Figure S2). Thus, when impairments are seen in locomotion and motor coordination, these will not be due to muscle weakness or reduced body size and weight.

#### 3.1.2. Open Field

In the open field test, motor activity and exploratory behavior of mice is commonly indicated by their distance moved, mean velocity and rearing on the hind limbs. As the center of an arena is an anxiogenic stimulus for mice, thigmotaxis (the tendency to stay and move along the wall of the arena) is used to measure anxiety and reaction to novelty. In this test, no differences were detected for mean velocity of L1/858–863 males and females and their corresponding WT animals. Velocity of males and females decreased with habituation to the novel environment, but differences were not significant (Supplementary Figure S3a,b).

**Figure 1:**
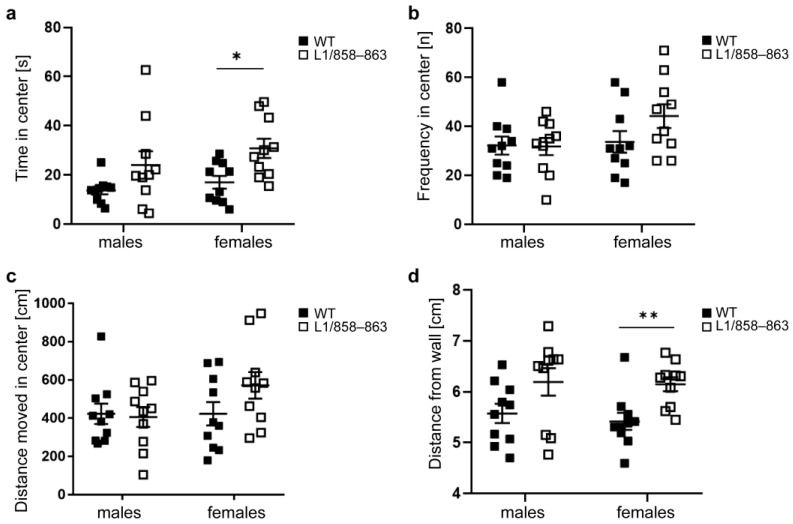
Mutant female, but not male mice spend increased time in the center zone and move to a higher distance from the wall in the open field. Mutant and WT males and females were subjected to the open field and the time spent in the center zone during the first 10 min (**a**), the frequency to enter the center zone during 20 min (**b**), the distance moved in the center during 20 min (**c**) and the distance from the wall during 20 min (**d**) were determined. Single values and average values ± SEM are shown; n = 10 mice per group; * *p* < 0.05, ** *p* < 0.01, two-way ANOVA with Tukey’s post hoc test.

Interestingly, time in the center zone in the first minutes of the test was longer for mutant females but not for mutant males compared to WT mice (Figure 1a). The frequency to visit the center zone was similar for mutant and WT males and females (Figure 1b). The total distance moved in the center zone was also similar for mutant and WT males and females (Figure 1c). The mean distance to wall was higher for the mutant females relative to WT females during the 20 test minutes, whereas it was similar for mutant and WT males (Figure 1d). No differences between genotypes were found for the other behavioral parameters analyzed such as stretched-attend posture and self-grooming (Supplementary Figure S4). Female mice of both genotypes performed more rearings on wall compared to off wall, while male mice did not show differences for rearings on wall compared to off wall (Supplementary Figure S4a). These results suggest reduced anxiety-like behavior or different reaction to novelty of L1/858–863 female mice, but not of L1/858–863 male mice.

#### 3.1.3. Elevated plus Maze

**Figure 2:**
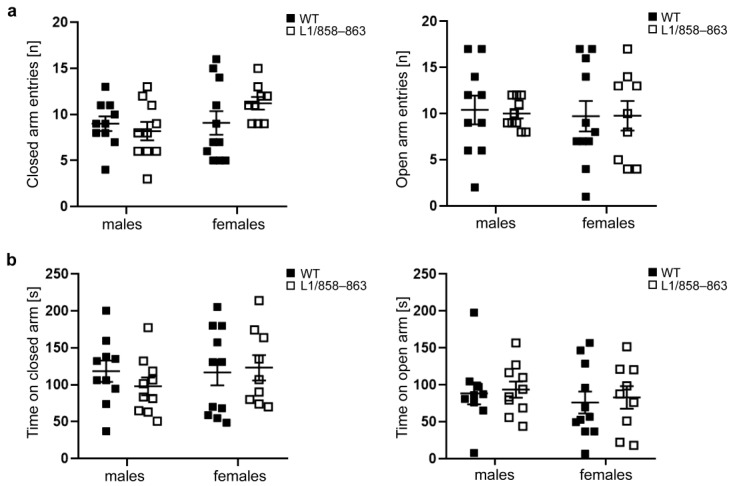
Mutant and WT mice do not show differences in anxiety states in the elevated plus maze. Mutant and WT males and females were subjected to the elevated plus maze to determine the open and closed arm entries (**a**) and the time spent in the open and closed arm (**b**). Single values and average values ± SEM are shown; n = 10 mice per group; two-way ANOVA and Tukey’s post hoc test.

To further compare exploratory and anxiety-like behavior of WT and transgenic mice the elevated plus maze was used. This test is based on the natural aversion of mice to open and elevated areas, as well as on their natural spontaneous exploratory behavior in novel environments. Mutant and WT mice did not exhibit differences in open and closed arm entries nor in time spent in the open and closed arms (Figure 2a,b). The ratio of the time spent in the open arms to the total time spent in the arena was 29 ± 15.49% for WT males, 31 ± 11.35% for mutant males, 28 ± 15.40% for WT females, and 25 ± 16.42% for mutant females. The ratio of the time in the closed arm compared to the total time was 39 ± 15.25% for WT males, 33 ± 12.55% for mutant males, 41 ± 17.04% for WT females and 39 ± 19.61% for mutant females, and the ratio of the time in the center compared to the total time was 31 ± 7.93% for WT males, 36 ± 10.13% for mutant males, 31 ± 5.55% for WT females and 36 ± 10.24% for mutant females. These results support the view that mutant mice display normal risk assessment and indicate that mutant and WT females have similar movement patterns in the elevated plus maze which stands in contrast to the altered behavioral patterns in the open field test.

#### 3.1.4. Social Interaction

**Figure 3:**
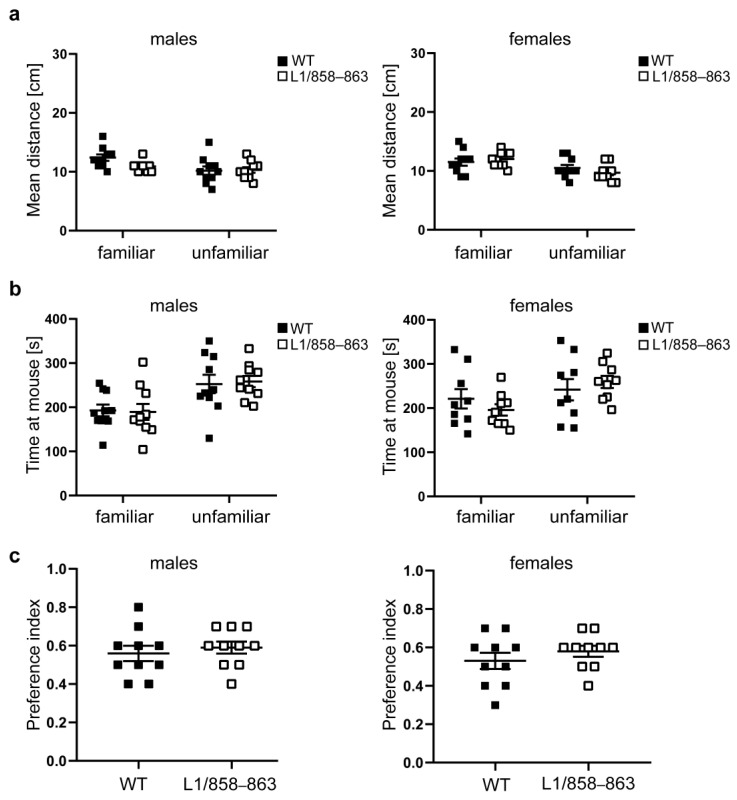
Unaltered social interest of mutants. Mutant and WT males and females were subjected to the social interaction test, and the distance to the familiar and unfamiliar mouse (**a**), and the time spent with the familiar and unfamiliar animal (**b**) was recorded for 10 min. The preference index was calculated (**c**). Single values and average values ± SEM are shown; n = 9–10 mice per group; two-way ANOVA and Tukey’s multiple comparison post hoc test (**a**,**b**) and Mann–Whitney test (**c**).

To assess memory and social interest, we performed the social interaction test, where mice tend to investigate more intensely unfamiliar mice than familiar ones. In this test, social preference is evaluated by measuring the time mice spent investigating familiar versus unfamiliar mice. Mutant and WT males and females kept the same distance to the familiar and unfamiliar mouse and remained only a slightly longer time at the unfamiliar mouse compared to the familiar mouse (Figure 3a–c). 

**Figure 4:**
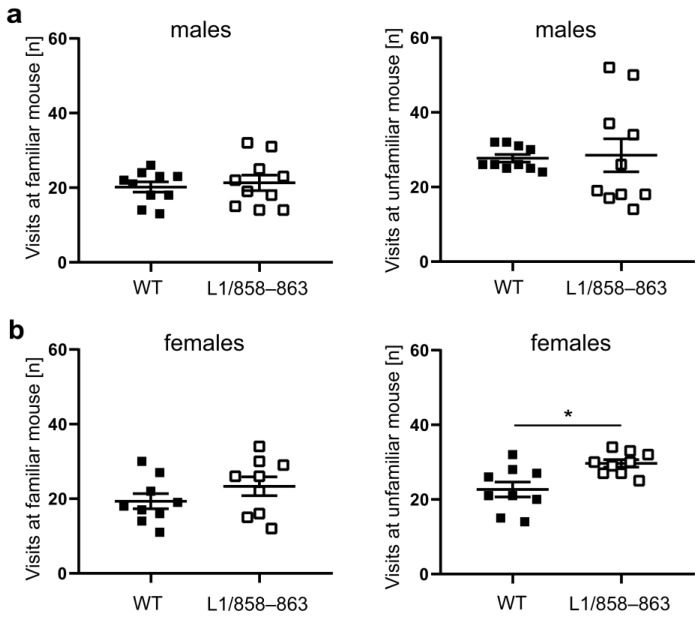
Social interest of mice of mutant and WT mice. Mutant and corresponding WT male and female mice were subjected to the social interaction test, and the frequency of visits of the familiar and unfamiliar mouse by males (**a**) and females (**b**) were determined during 10 min of the test. Single values and average values ± SEM are shown; n = 9–10 mice per group; * *p* < 0.05; Mann–Whitney test.

The frequency of visits at the familiar and unfamiliar mouse was similar for mutant and WT males, whereas mutant females visited the unfamiliar mouse more often than the WT females (Figure 4).

#### 3.1.5. Circadian Activity

**Figure 5:**
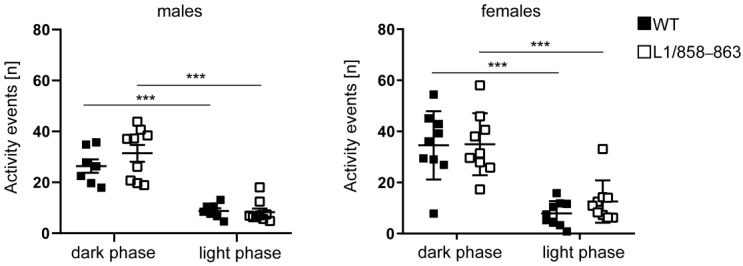
Mutant mice are normal in circadian rhythm and spontaneous activity. Activity of mutant and WT males and females was recorded in the home cage for 24 h every 4 min. Single values and average values ± SEM are shown; n = 8–9 per genotype; *** *p* < 0.001; two-way ANOVA and Tukey’s multiple comparison post hoc test.

To determine if mice display normal circadian rhythms and normal activity levels, circadian activity of single-housed mice was measured for 24 h. All mice were more active during the dark-phase compared to the light-phase (Figure 5) and male and female mutants were similar in activity compared to WT males and females, demonstrating that the mutant mice are normal in their circadian rhythm and in spontaneous behavior in the home cage.

#### 3.1.6. Accelerating Rotarod and Pole Tests

**Figure 6:**
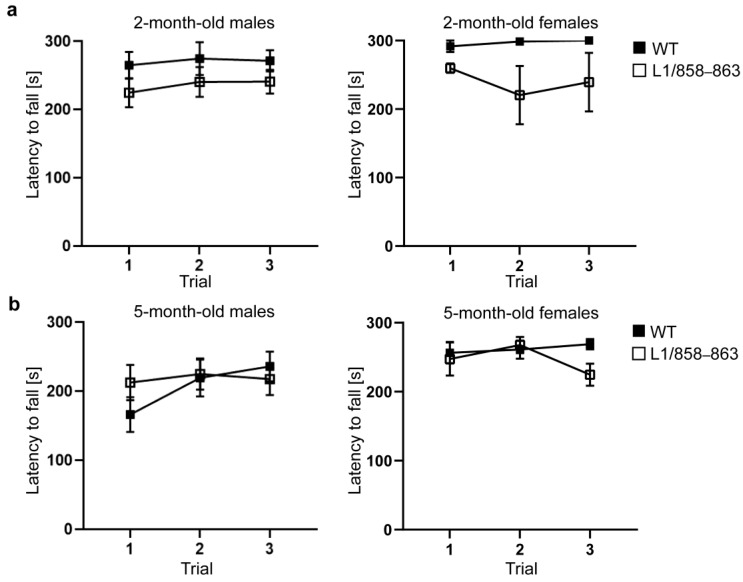
Normal motor performance of mutant mice in the rotarod test. Two-month-old male WT and mutant mice (**a**) and five-month-old male and female WT and mutant mice (**b**) were analyzed, and the latency to fall from the rotarod in three consecutive trials was determined. Average values ± SEM are shown; n = 10 for 5-month-old mice per group, n = 6 for 2-month-old mice per group; two-way repeated measures ANOVA and Tukey´s multiple comparison post hoc test.

L1-deficient mice and L1-deficient mice transduced to express mutated L1 which cannot be cleaved to produce the 70 kDa L1 fragment spend a shorter time on the accelerating rotarod compared to WT mice and L1-deficient mice transduced to express WT L1 [12]. Thus, we examined whether L1/858–863 mice are impaired in motor performance. Two-month-old and five-month-old male and female mice were analyzed. On the accelerating rotarod, mutant male and female mice spent the same time on the rotarod as WT males and females (Figure 6). Interestingly, the two-month-old WT males and females spent longer times on the rotarod compared to their five-month-old WT mice. In the pole test, no differences between the genotypes were seen. Moreover, the time to climb down and ability to turn 180° was similar (Supplementary Figure S5). These results indicate that motor coordination of mutant mice versus WT mice is not impaired.

#### 3.1.7. Marble Burying

**Figure 7:**
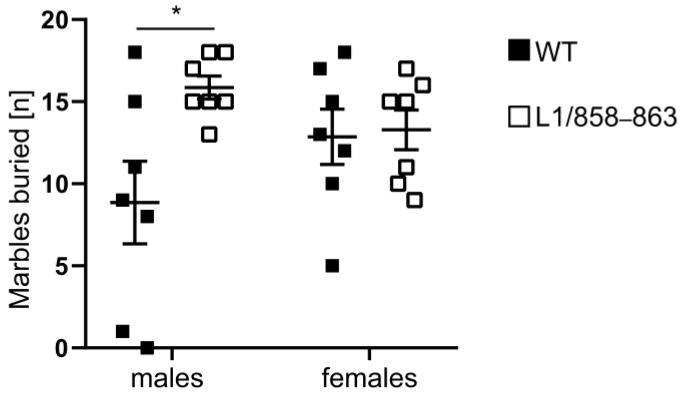
Mutant males show increased marble burying activity. Mutant and WT male and female mice were placed into a cage containing 20 black marbles and the number of marbles buried within 30 min was counted. Single values and average values ± SEM are shown; n = 7 mice per group; * *p* < 0.05; two-way ANOVA and Tukey’s post hoc test.

Previous studies had observed that heterozygous L1-deficient female mice exhibit an increased density in neurons in the neocortex and basal ganglia which could be indicative for an autistic-like phenotype [46]. Furthermore, L1 was shown to interact with methyl CpG binding protein 2 (MeCP2) [47] and mutations in MECP2 cause the Rett syndrome, which is an X-linked autism-spectrum disorder [48]. This suggests that mutations in L1 also cause autism-spectrum disorder phenotypes. Thus, we performed the marble burying test with mutant and WT males and females to evaluate digging activity that could suggest repetitive and compulsive-like behaviors or changes in anxiety and activity and object recognition. Mutant males buried more marbles than WT males, whereas female mutants buried the same number of marbles as WT females (Figure 7). These results indicate that, especially in males, the dibasic sequence mutation in the third FNIII region of L1 leads to enhanced digging behavior that could indicate obsessive-like behavior or altered activity and anxiety.

### 3.2. Histological Analysis

**Figure 8:**
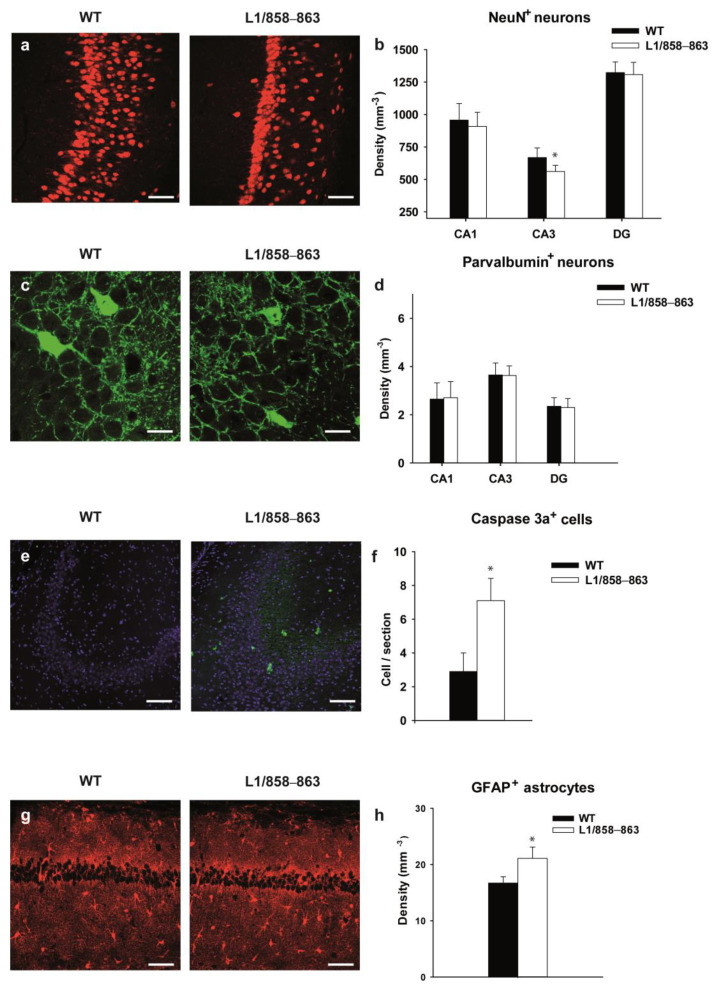
Histological analyses of male and female mutant and WT hippocampus. (**a**) Representative images of the NeuN-stained CA3 subfield. Scale bars, 50 µm. (**b**) Mean + SD of the densities of NeuN^+^ neurons in the CA1, CA3 and DG. Asterisk indicates a significant difference (*p* < 0.05; *t*-test, n = 6 mice per genotype). (**c**) Representative images of parvalbumin-stained interneurons in the CA3 subfield. Scale bars, 20 µm. (**d**) Mean + SD of the densities of parvalbumin^+^ neurons in the CA1, CA3 and DG. There was no significant difference between genotypes (*p* > 0.05; *t*-test, n = 6 mice per genotype). (**e**) Representative images of activated caspase-3a-stained (green) CA3 subfield. Scale bars, 50 µm. Cell nuclei are counterstained with DAPI (blue). (**f**) Mean + SD of the number of caspase 3a^+^ cells per section. Asterisk indicates a significant difference (*p* < 0.05; Mann–Whitney test, n = 6 mice per genotype). (**g**) Representative images of glial fibrillary acidic protein (GFAP)-immunostained hippocampus. Scale bars, 50 µm. (**h**) Mean + SD of the densities of GFAP^+^ astrocytes in the hippocampus. Asterisk indicates a significant difference (* *p* < 0.05; *t*-test, n = 6 mice per genotype).

Previous studies on L1-deficient mice and mice carrying the mutation D201N, which corresponds to the human disease-causing mutation D202N, revealed several morphological abnormalities in their brains [28,29,49,50]. Based on these findings, the brains of mutant mice were investigated for histological abnormalities. Using cell-type specific immunohistochemical markers, we investigated the hippocampus and motor cortex, as the structures underlying behavioral deficits. We separately investigated three males and three females per group (Supplementary Figure S6) and then combined the data, since there were no sex-dependent differences (Figure 8). We discovered several morphological changes in the hippocampus of mutants, whereas the motor cortex of mutant and WT mice was similar. First, we measured the area of several hippocampal subfields. The CA1, CA3 and dentate gyrus (DG) were similar in size for the genotypes (1.3781 ± 0.19 mm^2^ vs. 1.2361 ± 0.09 mm^2^ for the CA1, 0.5004 ± 0.07 mm^2^ vs. 0.4504 ± 0.05 mm^2^ for the CA3 and 0.7892 ± 0.09 mm^2^ vs. 0.7508 ± 0.05 mm^2^ for the DG in WT vs. mutant mice). We next counted the number of NeuN-positive neurons. The number of principal neurons was similar in the CA1 and DG in all genotypes, whereas mutant mice had a lower number of principle neurons in the CA3 subfield (−16%; *p* = 0.016, *t*-test, n = 6 mice per genotype; Figure 8a,b and Supplementary Figure S6a). To investigate inhibitory interneurons we used parvalbumin, a marker of fast spiking basket cells in the hippocampus, which comprise approximately 40% of all interneurons [51], and found no difference between the WT and mutant mice (*p* = 0.86; *p* = 0.64; *p* = 0.82, *t*-test for CA1, CA3 and DG, respectively; n = 6 mice per genotype; Figure 8c,d and Supplementary Figure S6b). 

To investigate whether the lower number of neurons is a consequence of apoptotic or necrotic cell death, we stained the hippocampi for activated caspase-3, as a marker of apoptotic cell death [46], and GFAP and ionized calcium-binding adapter molecule 1 (Iba-1) as markers for astrocytes and microglia, respectively. Since only very few activated caspase-3 expressing cells were detectable, the numbers of all hippocampal cells immunostained per section were counted (Figure 8e and Supplementary Figure S6d). Higher numbers of activated caspase-3-positive apoptotic cells were seen in mutant vs. WT hippocampi (*p* = 0.005, Mann–Whitney test; n = 6 mice per genotype; Figure 8f and Supplementary Figure S6c). Additionally, the number of GFAP immunopositive astrocytes was also higher in mutant than in WT hippocampi (*p* = 0.006, *t*-test, n = 6 mice per genotype; Figure 8g,h and Supplementary Figure S6d). The numbers of microglia were similar in all genotypes (Supplementary Figure S6e). We conclude that the number of pyramidal neurons, but not interneurons is decreased in mutant vs. WT mice, and that there is increased apoptotic cell death accompanied by astrogliosis in mutant mice.

**Figure 9:**
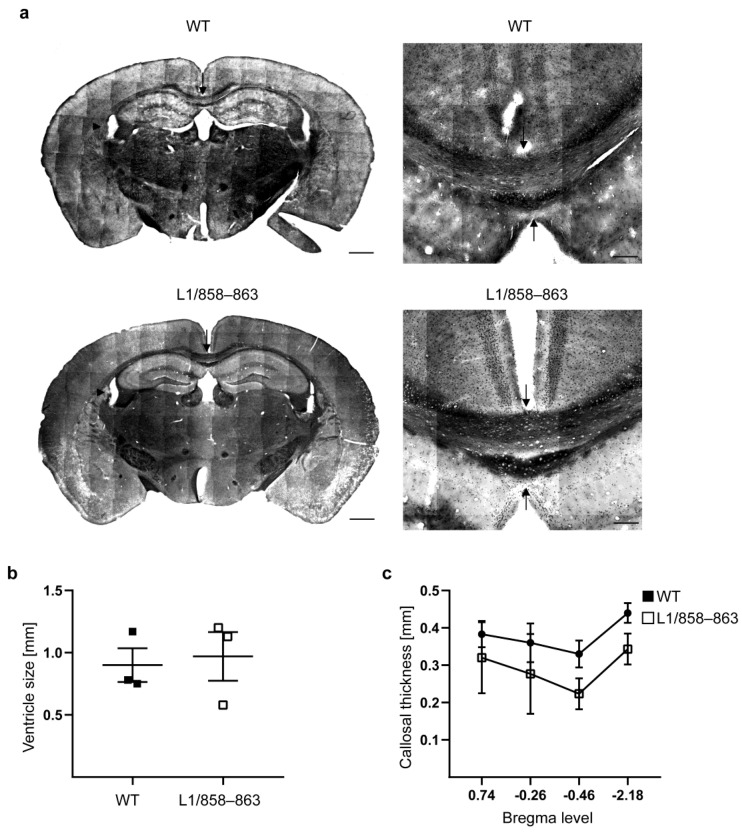
Width of the corpus callosum and ventricle size are normal in mutant mice. Coronal sections from brains of mutant and WT male mice were stained with Luxol Fast Blue and cresyl violet. (**a**) Representative overview images of brains (left) and the corpus callosum (right). Scale bars, 250 µm (right) and 500 µm (left). Arrows point to the corpus callosum, and arrowheads point to the ventricles. Graphs show ventricle size at Bregma 0.74 (**b**) and width of the corpus callosum at different Bregma levels and the midline (**c**). Average values ± SEM are shown; n = 3–4 mice per group; two-way ANOVA with Tukey´s post hoc test for corpus callosum width and Mann–Whitney test for ventricle size.

Furthermore, we determined the width of the corpus callosum and the size of the ventricles in male brains since a reduction in the width of the corpus callosum and an enhancement of the size of the ventricles were found in L1-deficient male mice [25,32], in male mice with the L1 p.D201N mutation (L1-201 mice) [26] and in patients with L1 syndrome [47]. Our results suggest that L1/858–863 mutant phenotypes are not due to alterations in the width of the corpus callosum or in ventricle size (Figure 9). Moreover, the gross architecture of the total brain appeared normal.

## 4. Discussion

Previous studies have shown that absence of L1 during brain development not only leads to altered brain anatomy and abnormal neural functions, but also to behavioral deficits. L1-deficient male mice exhibited an impaired exploratory behavior and stereotype circling behavior [52]. These mice avoided the center of an open field and had no interest in female mice in a social interaction paradigm. Furthermore, L1-deficient male mice displayed deficits in locomotor functions as well as weak and uncoordinated hind limbs [12,28,53]. In mice with conditional ablation of L1 in adulthood and L1 expression during nervous system development, we had observed decreased anxiety, altered place learning and increased CA1 basal excitatory synaptic transmission [54]. Moreover, heterozygous female L1-deficient mice were found to express an autism-like phenotype, to contain increased numbers of neurons and to have an enhanced metabolism in the forebrain [33,46]. Mice carrying a mutation found in human individuals with L1 syndrome exhibited brain malformations and impaired motor coordination [29]. In contrast, transgenic mice expressing L1 in astrocytes showed increased flexibility and selectivity in spatial learning [11]. Recently, we had generated gene-edited mice expressing full-length L1 but lacking the 70 kDa L1 fragment [34], which is transported into nuclei and mitochondria and had been suggested to play a role in motor functions [12]. In these mice, full-length L1 reached the cell surface and generation and localization of L1-fragments not generated by myelin basic protein were normal [34]. Analysis of cultured cerebellar granule cells from these mice revealed impaired neurite outgrowth and neuronal survival as well as impaired mitochondrial functions [55]. Based on these observations and the knowledge of the importance of L1 fragments for cellular functions [12,16,17,25,55,56], we hypothesized that mutation in the dibasic sequences exchanging wild-type RKHSKR for mutant SKHSSS in the third FNIII region of L1 would also affect mouse behavior and development of brain architecture.

Here, we show that mutant mice exhibit specific changes in behavior. Female mutants stayed a longer time in the center of the open field and at a higher distance from the wall, which could suggest reduced anxiety or reaction to novelty. Interestingly, male mutants did not differ from the WT males in the open field test. In addition, open and closed arm entries and risk assessment in the elevated plus maze were similar for mutant and WT males and females, showing normal anxiety and reaction to novelty of males. Although female mutants visited the unfamiliar mouse more often in the social interaction test, other parameters such as time spent with the familiar and unfamiliar mouse, as well as distance to the familiar and unfamiliar mouse, were comparable to WT females. Mutant and WT males had the same preferences in the social interaction test. No changes in circadian activity were seen in the male and female mutant and WT mice and motor performance in the rotarod test, the beam-walking test and the pole test were normal. Interestingly, male L1/858–863 mice, but not female mutants, displayed enhanced activity in the marble burying test but no changes in self-grooming. Digging behavior belongs to the normal behavior repertoire of mice and alterations seen in the marble burying test could suggest anxiety- and compulsive-like behavior or enhanced activity [57,58]. Since general circadian activity of mutants was normal and distance moved and velocity in the open field test were similar to that of WT males, it is unlikely that the enhanced digging behavior is a sign for enhanced activity of male mutants. In addition, transgenic male mice did not show enhanced anxiety in the open field and elevated plus maze tests. It is thus possible that the enhanced digging behavior relates to an obsessive-like phenotype of male mutants. For a more detailed assessment of mutant symptoms of obsession or compulsion, further tests will be needed.

In the context of the present study, we would like to mention that L1-70 interacts with topoisomerase 1 and disruption of this interaction leads to changes in expression of the long autism genes neurexin 1 (Nrxn1) and neuroligin 1 (Nlgn1) [56]. Of note, cortical neurons from L1/858–863 mutant mice contained lower mRNA levels of these two genes, which participate in synapse development and synapse maintenance as well as modulation of glutamatergic synaptic function in the central nervous system, which are associated with neuropsychiatric disorders such as autism spectrum disorders [59,60,61,62,63,64]. Thus, the reduced expression of Nrxn1 and Nlgn1 in mutant mice could contribute to the changes in anxiety and activity, as we report here. In addition, the third FNIII domain and especially the dibasic sequences within RKHSKR, which are mutated in the L1/858-863 mice, appear to be involved in cell adhesion and neurite outgrowth and to control L1 homo-multimerization and binding of L1 to integrin β1 [14,65,66,67,68]. Therefore, the mutation of the dibasic sequences in mutant mice could not only alter proteolytic cleavage of L1, but also L1 homophilic interactions and interaction with integrins that could contribute to the alterations in behavior and death of certain principle neurons in the hippocampus.

Furthermore, we observed differences in behavior of mutant male and female mice. This suggests that the mutation in the third FNIII domain of L1 and differences in the levels of sex hormones or differences in brain structure of male and female mice affect their behavior. The mutation in the dibasic sequences in the third FNIII domain could alter L1 signaling or interactions of L1 with other proteins, e.g., in the nucleus, leading to changes in gene expression which differently modulate behavior of males and females. In previous studies we observed that L1 interacts with the estrogen receptors α and β, the retinoid-X-receptor and peroxisome proliferator-activated receptor γ (PPARγ) and showed that the interaction of L1 with PPARγ and topoisomerase 1 is mediated by L1-70 [12,56]. Thus, we speculate that the mutant phenotype results from changes in functions of Nrxn1, Nlgn1, PPARγ and topoisomerase 1, which contribute to the changes in behavior. Since we did not see differences in hippocampal morphology of male and female mice, environmental or other factors could contribute to the differences observed in behavior of males and females.

Our results are in agreement with the findings that the multiple mutations in L1 lead to different disease outcomes with different severity in humans [69,70,71] (see also http://www.l1cammutationdatabase.info/; access date 10 January 2023). Several mutations in the FNIII-like domains are disease-causing in humans (see for instance: [71,72,73,74,75]). Some of these disease-causing mutations were found in the vicinity of sequences that are mutated in the L1/858–863 mice. A patient with p.Arg846His mutation of L1, which is close to the position mutated in the L1/858–863 mice, displayed childhood onset hyperactivity, psychosis and autism spectrum disorder with symptoms overlapping with those seen in obsessive–compulsive disorders [74]. Interestingly, in mice treated with lipopolysaccharide, social withdrawal and increased stereotype activity in males was linked to a reduced number and volume of Reelin expressing cells, to a reduced expression of trans-synaptic cell-adhesion molecules and to alterations in spine density [76]. Although only male L1/858–863 mice exhibited increased digging activity which could suggest sex-dependent enhanced activity or increased stereotype behavior, male and female mutant mice did not show sex-dependent alterations in brain morphology or differences in numbers of principle cells in the hippocampus. Sex-dependent changes in neuronal activity as well as synapse shape and density could nonetheless be responsible for the differences in the behavior of mutant males and females and will be a topic of future research.

It was proposed that development of a mild-to-moderate hydrocephalus could lead to structural brain deficits especially in the middle temporal and middle frontal gyrus prior to the behavior changes [77]. In human patients, corpus callosum size and impaired inter-hemispheric communication channels necessary to sustain motor control and attention were demonstrated to influence attention-deficit and hyperactivity in adulthood [78]. Enlarged lateral ventricles, reduced size of the corticospinal tract and of the corpus callosum as well as errors in corticospinal axon guidance were observed in L1-deficient mice [28,49,52]. Mice with L1 p.D201N mutation also developed a marked hydrocephalus and exhibited reduced callosal width [29]. Interestingly, mice expressing L1 with a deletion of the sixth Ig-like domain showed normal brain development, normal development of the decussation of the corticospinal tract, of the thalamocortical tract, of the corpus callosum as well as normal anatomical features when outbred to the 129/Sv background, but a severe hydrocephalus on the C57BL/6 genetic background [79]. Of note, female heterozygous L1-deficient mice display an autism-like phenotype [33] and L1-deficient male mice are impaired in motor coordination [12]. Rats infused with antibodies neutralizing L1 displayed deficits in spatial learning [80], which implicates a role for L1 in the regulation of mechanisms that underlie the predisposition to exhibit mood and anxiety disorders in adulthood [81]. Mice with the L1/858-863 mutation investigated here had normal-sized ventricles and a normal corpus callosum, suggesting that the mutated region of the third FNIII domain of L1 is not required for normal corpus callosum development. Additionally, in mutant mice, we observed a reduced number of principle neurons in the CA3 region of the hippocampus and enhanced neuronal death and activation of astrocytes, but no changes in activation of microglia. Since a smaller hippocampus with fewer pyramidal and granule cells was also seen in L1-deficient mice [82], we hypothesize that the reduction in principle cell number in the hippocampus of L1/858–863 and L1-deficient mice is due to the absence of L1-70. We observed reduced mitochondrial complex I activity, reduced mitochondrial membrane potential and enhanced retrograde transport of mitochondria in cultured L1/858–863 cerebellar neurons [55]. Similar mitochondrial dysfunctions may lead to the loss of principle cells and an enhanced number of caspase-3 immunostained cells in the hippocampus of L1/858–863 mice. Mitochondrial dysfunctions are known to contribute to the development of neurodegenerative diseases [83,84]. Mitochondria sense and integrate psychosocial and behavioral factors and transduce them into cellular and molecular modifications, thereby contributing to different types of psychological states [85]. Mitochondrial dysfunctions were also observed in L1-deficient cerebellar neurons. L1-deficient neurons had a reduced mitochondrial complex I activity and membrane potential, reduced mitochondrial velocity and enhanced retrograde transport [25]. We thus propose that mitochondrial dysfunction of mutant neurons may result in neuronal cell loss, astrogliosis and changes in behavior.

To conclude, L1/858–863 mice exhibited higher numbers of caspase-3 positive cells and astrogliosis, displayed reduced anxiety and male mutant mice showed an enhanced burying behavior, while motor functions and circadian activity were normal. Mitochondrial dysfunction, deficits in L1 signaling and L1 interactions leading to impaired survival and outgrowth of neurons as well as reduced neurexin/neuroligin signaling or alterations in synapse shape and density could underlie the observed changes in the mutant mice. Furthermore, environmental factors could be important in the manifestation of the phenotype of the mutant mice since the behavioral analysis unexpectedly revealed only very small differences and although sex-dependent differences were observable in behavior, we did not see differences between sexes in immunohistochemistry. These environmental factors could also contribute to the development of the L1 syndrome in patients influencing the severity of the disease.

## 5. Conclusions

Our findings suggest that proper functioning of the cell adhesion molecule L1 is of importance for normal behavior and that mutations in the third FNIII domain of L1 can contribute to development of neurological disorders. These mutations can cause changes in specific brain regions and neuronal activity, which then affect behavior.

## Data Availability

Data and materials will be made available upon reasonable request.

## Supplementary Materials

The following supporting information can be downloaded at: https://www.mdpi.com/article/10.3390/biom13050776/s1, Figure S1: Schematic presentation of L1 in mice expressing wild-type L1 or L1 with RKHSKR/SKHSSS mutation; Figure S2: Normal quadriceps muscle function in L1/858–863 mice; Figure S3: Normal velocity and total distance moved of L1/858–863 mice in the open field; Figure S4: Unaltered stretched-attend posture, self-grooming and rearing on and off wall by L1/858–863 mice in the open field; Figure S5: Normal motor performance of L1/858–863 mice in the pole test; Figure S6: Histological analyses of the L1/858–863 and wild-type (WT) hippocampus; Figure S7: Timeline of behavioral experiments.
