# Supplementary material for: Mice Mutated in the Third Fibronectin Domain of L1 Show Enhanced Hippocampal Neuronal Cell Death, Astrogliosis and Alterations in Behavior
Source: Biomolecules. 2023 Apr 29;13(5):776. doi: 10.3390/biom13050776 (PMC10216033; doi:10.3390/biom13050776)
Supplement: Supplementary file 1 [file biomolecules-13-00776-s001.zip › biomolecules-2331215-supplementary.pdf]

Supplementary Material

Ludovica Congiu, Viviana Granato, Igor Jakovcevski, Ralf Kleene, Luciana Fernandes, Sandra Freitag, Matthias Kneussel, Melitta Schachner, Gabriele Loers: Mice mutated in the third fibronectin domain of L1 show enhanced hippocampal neuronal cell death, astrogliosis and alterations in behavior.

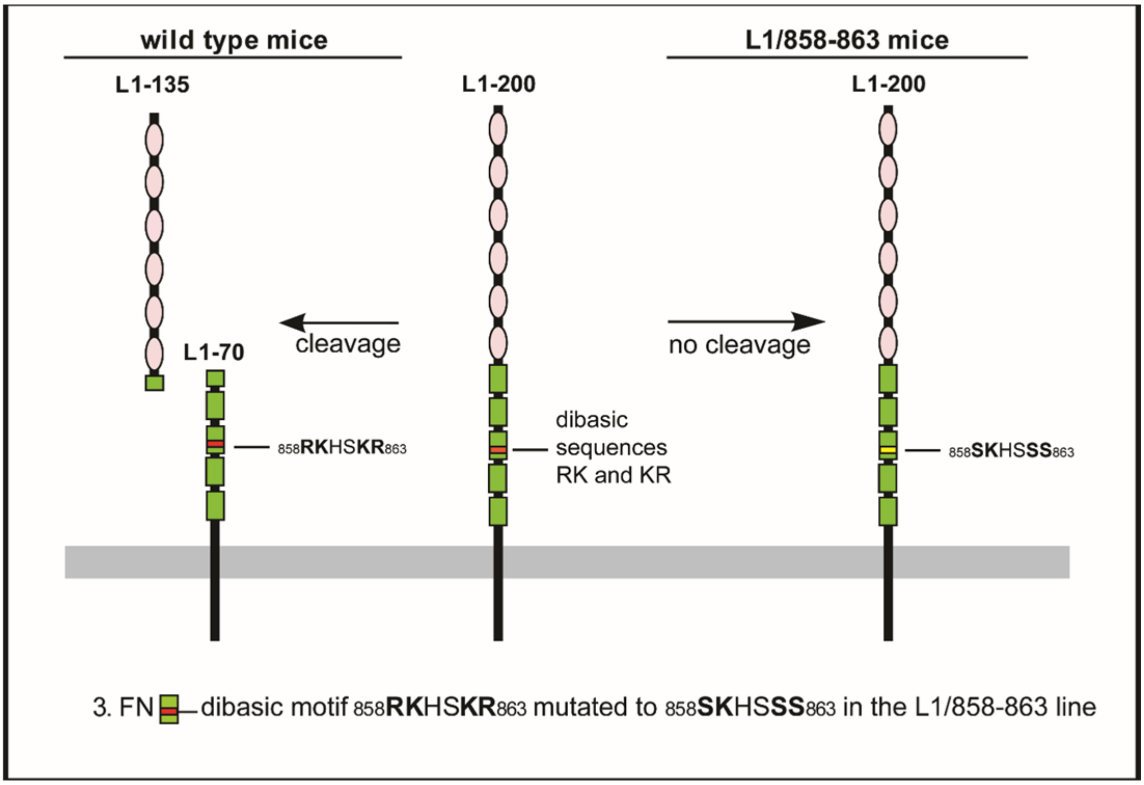

Supplementary Figure S1: Schematic presentation of murine L1 expressing wild type L1 or L1 with a RKHSKR to SKHSSS mutation. L1 consists of six immunoglobulin-like domains (ovals) and five fibronectin type III homologous repeats (rectangles) in the extracellular domain, a transmembrane domain passing the plasma membrane and an intracellular tail. L1 is cleaved in its third FNIII domain at the dibasic sequence RK or KR within $_{858}$RKHSKR$_{863}$ by the serine proteases PC5a, plasmin and trypsin. Mutation in this domain converting $_{858}$RKHSKR$_{863}$ to $_{858}$SKHSSS$_{863}$ (L1/858-863 mutant mice) prevents the generation of L1-70 and L1-135.

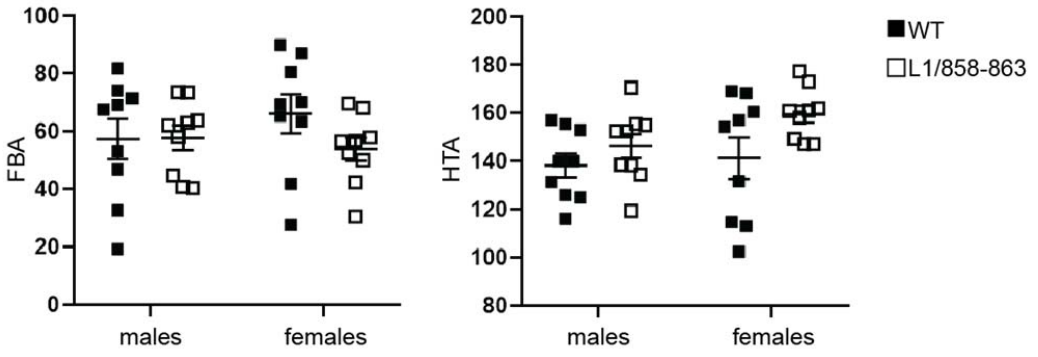

Supplementary Figure S2: Unaltered quadriceps muscle function in L1/858-863 mice. Male and female L1/858-863 and corresponding wild type (WT) mice were subjected to the beam walking test, and foot-base-angle (FBA) and heals-tail-angle (HTA) were determined. Single values and average values ± SEM are shown; n = 8-10 mice per group; two-way ANOVA with Tukey´s post hoc test.

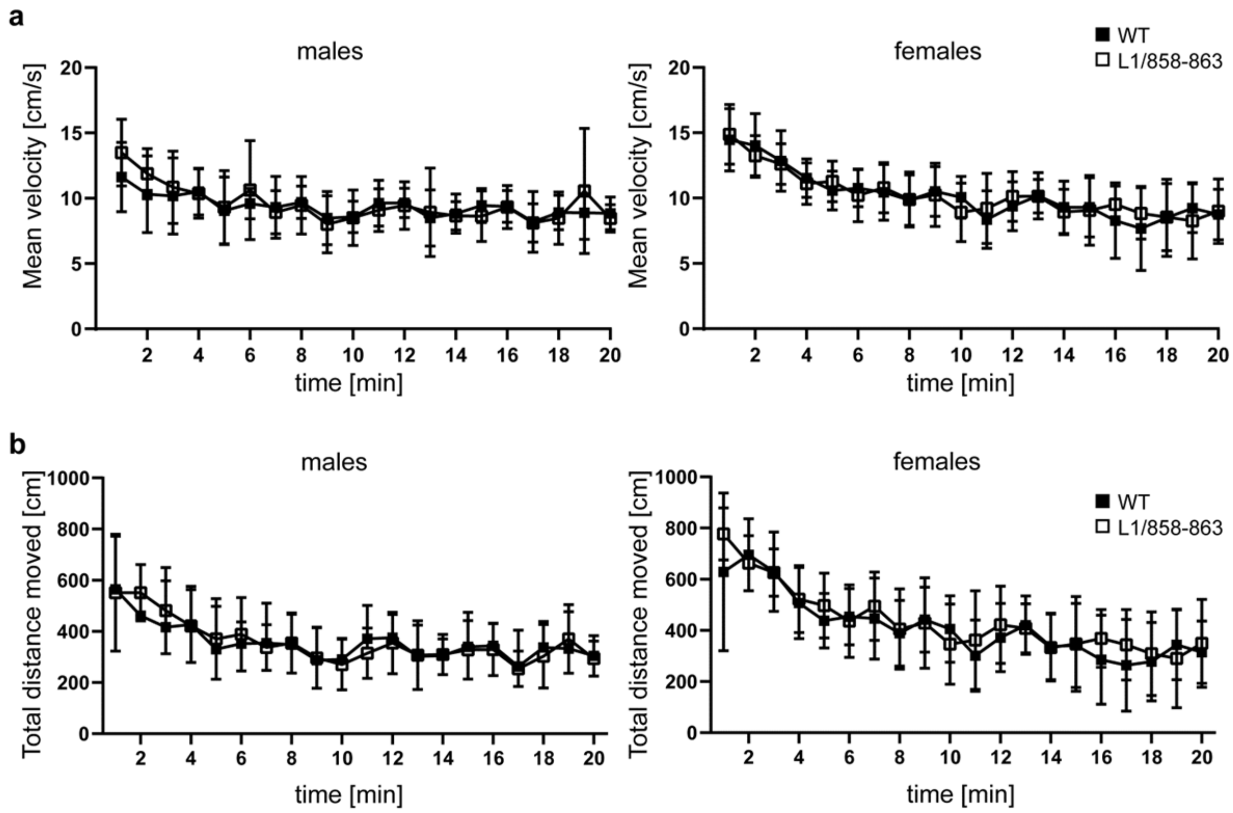

Supplementary Figure S3: Unaltered velocity and total distance moved by L1/858-863 mice in the open field. Male and female L1/858-863, and corresponding wild type (WT) mice were subjected to the open field test and the velocity (a) and distance moved (b) were determined over 20 min. Average values ± SD are shown; n = 10 mice per group; two-way repeated measures ANOVA with Tukey´s post hoc test.

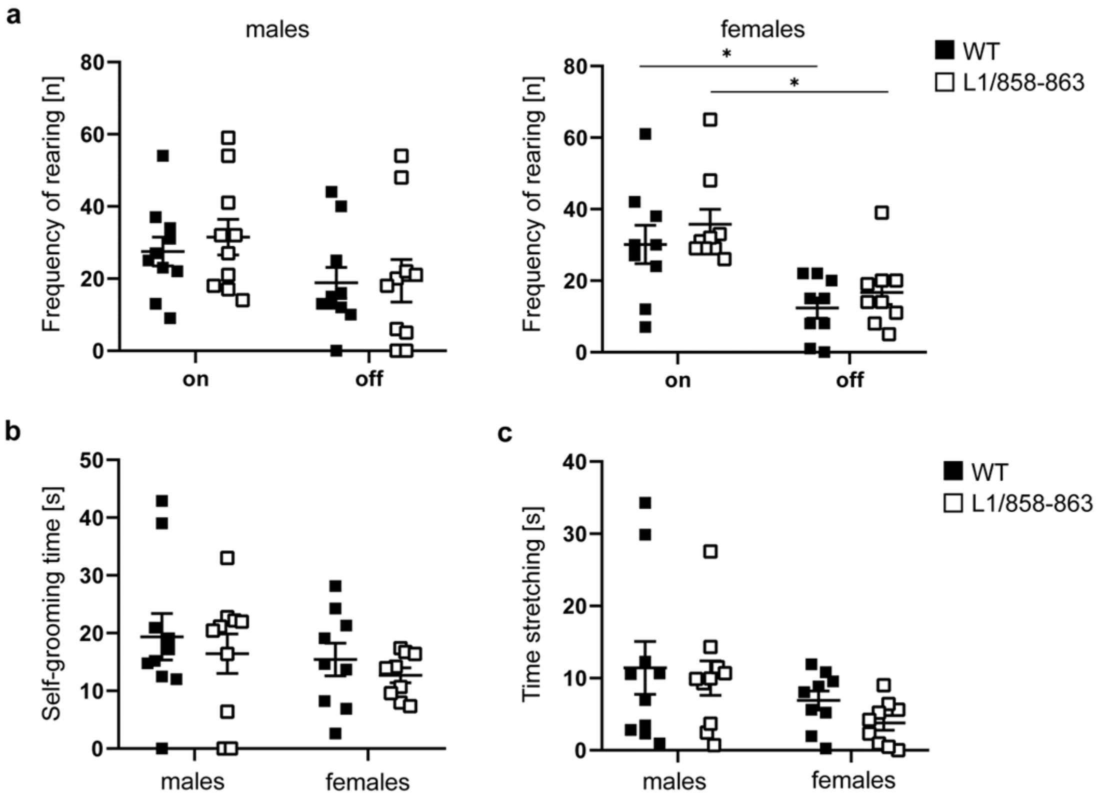

Supplementary Figure S4: Unaltered stretched attend posture, self-grooming, and rearing on and off wall by L1/858-863 mice in the open field. L1/858-863 and corresponding wild type (WT) male and female mice were subjected to the open field test and the number of rearings on wall (on) and off wall (off) (a) time spent self-grooming (b) and time with stretched attend posture (c) were determined over 20 min. Average values ± SD are shown; n = 9-10 mice per group; * $p < 0.05$, two-way ANOVA with Tukey´s post hoc test.

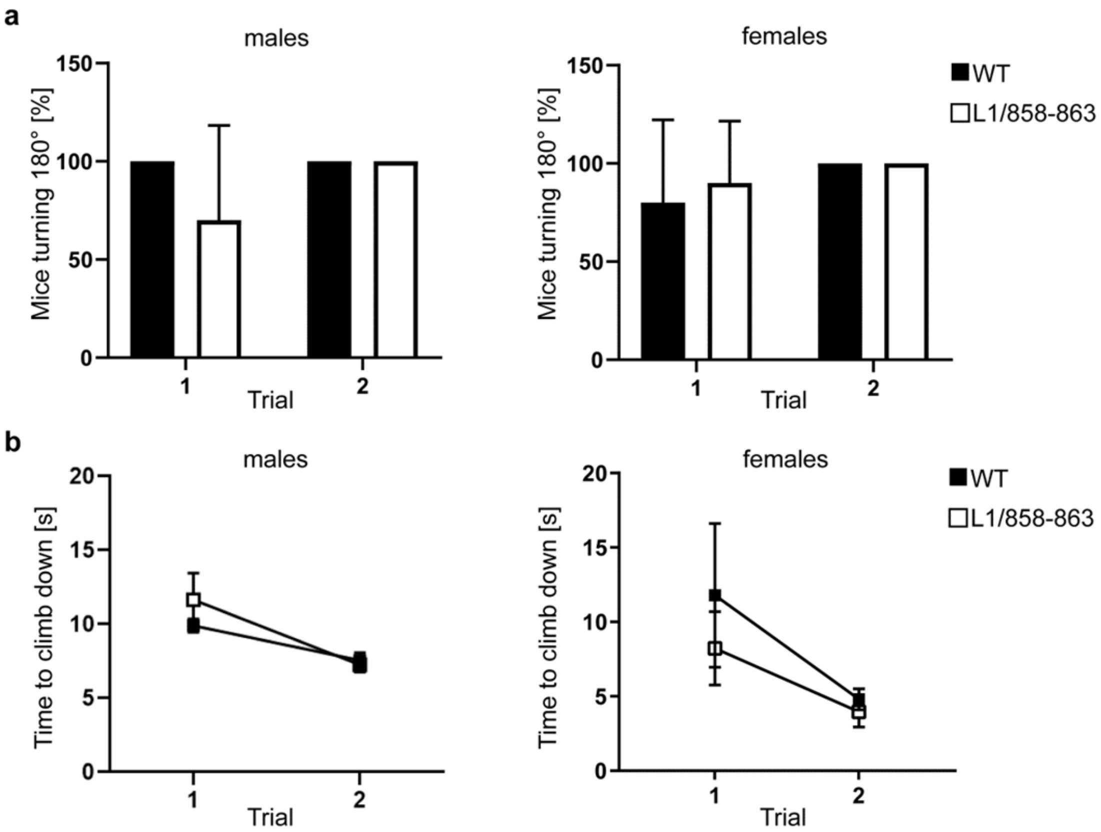

Supplementary Figure S5: Unaltered motor performance of L1/858-863 mice in the pole test. L1/858-863 and corresponding wild type (WT) male and female mice were subjected to the pole test, and the ability to turn 180° (a) and time needed to climb down (b) in two consecutive trials were measured. Average values ± SD (a) or SEM (b) are shown for the percentage of mice turning 180° and time to climb down; n = 10 mice per group; two-way ANOVA and Tukey´s multiple comparison post hoc test.

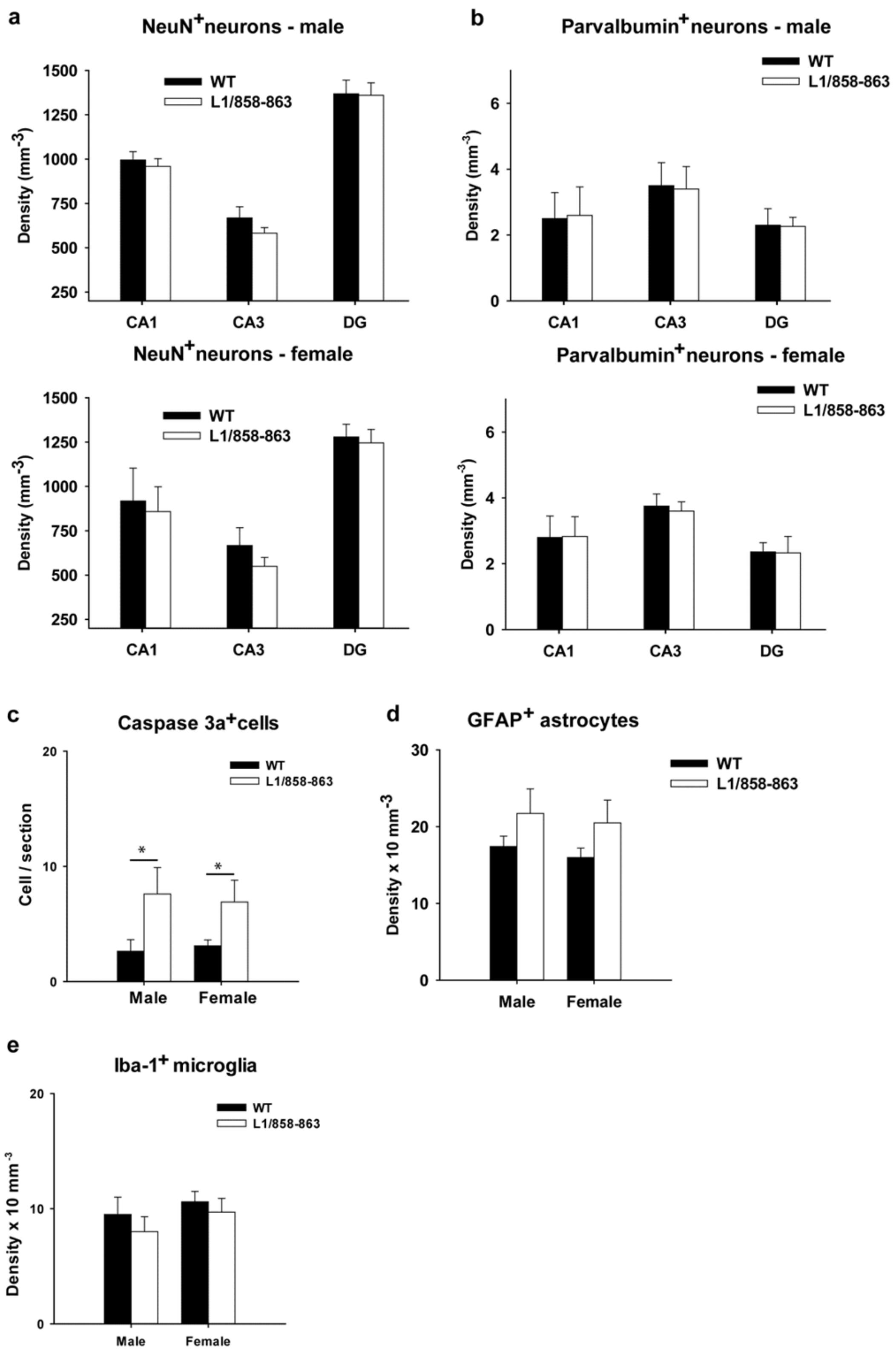

Supplementary Figure S6: Immunohistological analyses of the L1/858-863 and wild type (WT) hippocampus. (a) Mean + SD of the densities of NeuN+ neurons in the CA1, CA3 and DG for male (upper

panel) and female (lower panel) mice. (b) Mean + SD of the densities of parvalbumin+ neurons in the CA1, CA3 and DG for male (upper panel) and female (lower panel) mice. (c) Mean + SD of the number of caspase 3a+ cells per section. (d) Mean + SD of the densities of GFAP+ astrocytes in the hippocampus. (e) Mean + SD of the densities of Iba+ microglia in the hippocampus. Asterisks indicate significant difference between genotypes ($p < 0.05$; t-test, n = 3 mice per genotype and sex).

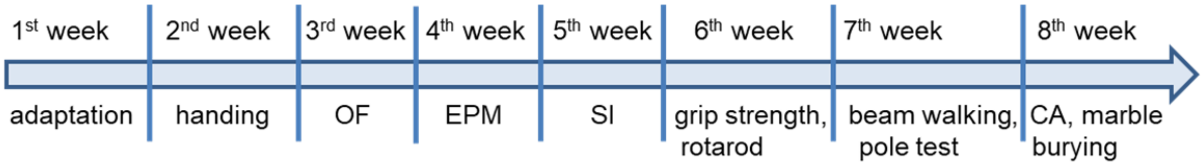

Supplementary Figure S7: Timeline of behavioral experiments. EPM: elevated plus maze, OF: open field, SI: social interaction, CA: circadian activity. Mice were housed singly for 3 days before recording the activity in the home cage.
